# Modified Negative Pressure Wound Therapy Using Surgical Drain and Occlusive Sutures for Leakage After Esophageal Injury

**DOI:** 10.1093/icvts/ivaf296

**Published:** 2025-12-04

**Authors:** Ho Seong Cho, Min Su Kim, Chiseung Lee, Hyo Yeong Ahn

**Affiliations:** Department of Thoracic and Cardiovascular Surgery, School of Medicine, Pusan National University, and Biomedical Research Institute, Pusan National University Hospital, Busan 49241, Republic of Korea; Department of Biomedical Engineering, Graduate School, Pusan National University, Busan 49241, Republic of Korea; Department of Biomedical Engineering, School of Medicine, Pusan National University, and Biomedical Research Institute, Pusan National University Hospital, Busan 49241, Republic of Korea; Department of Thoracic and Cardiovascular Surgery, School of Medicine, Pusan National University, and Biomedical Research Institute, Pusan National University Hospital, Busan 49241, Republic of Korea

**Keywords:** esophageal injury, negative pressure wound therapy, anastomotic leakage

## Abstract

Oesophageal leakage is a serious complication that can lead to infection, sepsis, malnutrition, and death. Negative Pressure Wound Therapy has been increasingly used to treat oesophageal leaks; however, in cases of cervical oesophageal leakage, deep tract and anatomical complexity often hinder an airtight seal and limit transmission of negative pressure. We developed a modified Negative Pressure Wound Therapy technique by connecting a surgical drain to the base of the foam dressing and securing it with subcutaneous occlusive sutures to ensure a reliable seal and effective negative pressure transmission. Four cases of modified application were included in this study. All leaks were controlled without complications attributable to the procedure. The modified Negative Pressure Wound Therapy technique effectively addresses the limitations of conventional methods in complex anatomical regions. By ensuring a reliable occlusive seal and enhancing negative pressure transmission, this approach promotes optimal wound healing through improved exudate clearance and reduced maceration.

## INTRODUCTION

Oesophageal leakage has multiple etiologies, especially due to anastomotic leakage or oesophageal injury. Anastomotic leakage following surgery is a serious complication, with an incidence ranging from 3% to 25% and a mortality rate between 17% and 35%.^1^^,^^2^ The leakage of corrosive digestive fluids can severely damage surrounding organs and tissues, leading to infection, sepsis, and malnutrition—all of which are associated with increased mortality.

Negative Pressure Wound Therapy (NPWT) is a well-established wound management modality known for its effectiveness in evacuating wound exudate, enhancing tissue perfusion, and promoting angiogenesis.^3^ Recently, innovative adaptations of NPWT have emerged, particularly for managing deep and narrow wounds.^4^

However, in cases of cervical oesophageal leakage, anatomical complexity often results in poor adherence of the dressing and inadequate delivery of negative pressure to the wound bed.^5^ Achieving an airtight seal around the foam dressing remains a technical challenge.

To address these limitations, we developed a modified NPWT technique by connecting a surgical drain to the base of the foam dressing and securing it with subcutaneous occlusive sutures to ensure a reliable seal and effective negative pressure transmission.

In this report, we present a series of cases in which this modified NPWT technique was successfully applied to treat anastomotic leaks following oesophageal cancer surgery.

## CASE PRESENTATION

### # Case 1

A 65-year-old male patient with a past medical history of diabetes mellitus, hypertension, and chronic kidney disease presented with a left pyriform sinus carcinoma invading the oesophagus. He subsequently underwent a total pharyngolaryngoesophagectomy with gastric pull-up.

On postoperative day (POD) 12, the patient developed leucocytosis. Esophagography performed at that time demonstrated an anastomotic leak. Initial management involved the application of conventional NPWT therapy on POD 14. We used CuraVAC (CGBIO Co., Ltd) for NPWT dressing and Curasys (CGBIO Co., Ltd) for NPWT pump. However, repeated air leakage led to frequent NPWT malfunctions, compromising the therapeutic effect.

Given these challenges, modified NPWT therapy was initiated on POD 18. Follow-up esophagography on POD 40 revealed no evidence of leakage. Consequently, the modified NPWT was removed, and the wound was successfully closed. The patient demonstrated an uneventful recovery thereafter and was discharged on POD 60.

### # Case 2

A 44-year-old woman with papillary thyroid carcinoma underwent total thyroidectomy with bilateral central neck dissection. An intraoperative oesophageal injury was repaired with multiple sutures. On POD 2, she developed leucocytosis and fever; esophagography confirmed a leak. Conventional NPWT was applied. On POD 15, esophagography showed no leak and NPWT was removed. On POD 20, the leak recurred after vomiting; on POD 21, modified NPWT was initiated. By POD 33, the wound was clean; modified NPWT was removed and the wound closed. Oral feeding resumed on POD 44; she was discharged the same day.

### # Case 3

A 57-year-old woman with hypertension, diabetes, and prior cerebral infarction presented with cervical infection due to a foreign body-related oesophageal perforation. On POD 0, antibiotics were started, the abscess drained, and modified NPWT was applied. On POD 17, modified NPWT was removed, and a sternocleidomastoid flap was used for coverage. Esophagography on POD 18 showed no leak; oral feeding began on POD 19. She recovered uneventfully and was discharged on POD 21.

### # Case 4

A 60-year-old man with hypertension presented after foreign body ingestion, causing a cervical oesophageal perforation. On POD 0, endoscopic removal was performed; antibiotics were started, the abscess drained, the tear sutured, and modified NPWT applied. Esophagography on POD 11 showed no leak. Modified NPWT was removed on POD 12; oral feeding was initiated, and he was discharged on POD 14.

## HOW-TO-DO-IT: MODIFIED NPWT TECHNIQUE

### Step 1 attaching nasogastric (NG) tube to the foam (Video 1)

Trim a piece of polyurethane foam dressing to appropriately fit the wound cavity. Insert a sterile NG tube into the foam and secure it at two equidistant points using 2–0 nylon sutures. The NG tube functions as the conduit for negative pressure delivery.

### Step 2 foam placement

Insert the prepared foam into the wound cavity, ensuring that the tip of the NG tube is positioned at approximately two-thirds of the wound depth. This allows optimal fluid drainage while avoiding direct contact with the wound base.

### Step 3 temporary wound closure and initiation of therapy

Once the cavity is fully packed with foam, place a semipermeable film between the wound edges to prevent epidermal adhesion during future dressing changes. Approximate the skin edges and close with interrupted 3–0 nylon sutures. Connect the external end of the NG tube to a vacuum source and initiate continuous negative pressure therapy. Suction was continuous at −125 to −150 mmHg, and dressings were typically changed every 3-4 days at the bedside while the patient was awake, without sedation.

#### Baseline characteristics and outcomes

The median duration of modified NPWT application was 15 days, with a median of 3.5 dressing changes. The median time from injury to the resumption of feeding was 21.5 days. One complication was reported: One patient developed an oesophageal stricture, which was presumed to be related to suture repair performed during the initial surgery. The stricture was successfully managed through endoscopic balloon dilatation (**Table 1**).

**Table 1.** Baseline Characteristics and Outcomes of Modified NPWT

Case	Age (y)	Sex	Cause	Previous treatment	Timing of modified NPWT	Duration (*d*)	Change (*n*)	After modified NPWT	Feeding resume (d)	Complication
**# 1**	65	M	Surgery	Conventional NPWT	After failure	22	4	Penrose drain	24	–
**# 2**	44	F	Surgery	Conventional NPWT, suture	After recurrence	13	4	-	44	Stricture
**# 3**	57	F	Foreign body	–	Immediate	17	3	Muscle flap	19	–
**# 4**	60	M	Foreign body	Suture	Immediate	12	3	-	12	-
					Median	**15**	**3.5**		**21.5**	**25%**

Abbreviation: NPWT, Negative Pressure Wound Therapy.

## DISCUSSION

Anastomotic leaks following surgery can result in severe complications such as infection, sepsis, and malnutrition, all of which are associated with high mortality. A structured, stepwise management approach is essential to control infection progression and ensure adequate nutritional support. Given the high viscosity of saliva and gastric secretions typically present in wound exudate, effective drainage and prevention of tissue maceration are critical for promoting wound healing.

NPWT offers an effective strategy to manage exudate and prevent maceration.^3^ However, its efficacy can be limited in anatomically complex regions such as the cervical oesophagus, where challenges in achieving an airtight seal often lead to dressing detachment and insufficient negative pressure transmission to the wound interface. Similar technical difficulties are encountered in tracheostomy wounds, where securing a reliable occlusive seal around the dressing is often problematic.

In deep cavitary wounds, conventional NPWT may be insufficient, as healing tends to occur superficially while fluid stagnates in the deepest areas. Likewise, cervical oesophageal leaks form narrow, elongated tracts where saliva and secretions frequently pool, further hindering wound healing despite NPWT application.

To address these limitations, we applied a modified NPWT technique involving the placement of a surgical drain at the base of the foam dressing, secured with subcutaneous occlusive sutures to maintain an effective seal. This approach offers 2 key advantages: (1) it minimizes the risk of non-occlusive dressing failure, and (2) it reduces the distance from the suction source to the wound bed, thereby enhancing negative pressure transmission and fluid evacuation.

**Figure 1.**
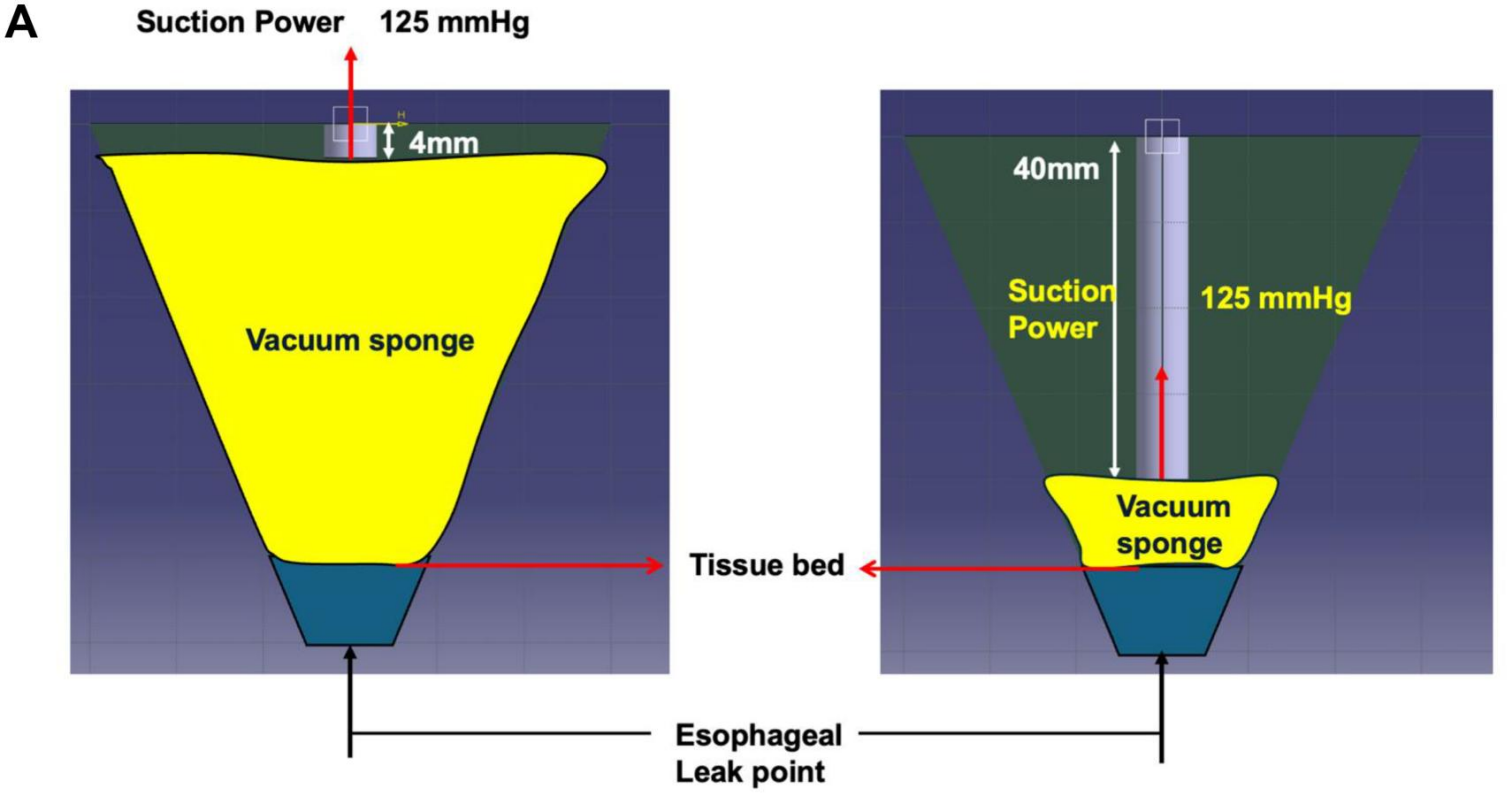
Schematic Illustration Comparing Conventional and Modified Negative Pressure Wound Therapy Techniques. The Modified Approach, Incorporating a Surgical Drain at the Base of the Foam Dressing, Enhances Negative Pressure Delivery to the Tissue Bed

**Figure 2.**
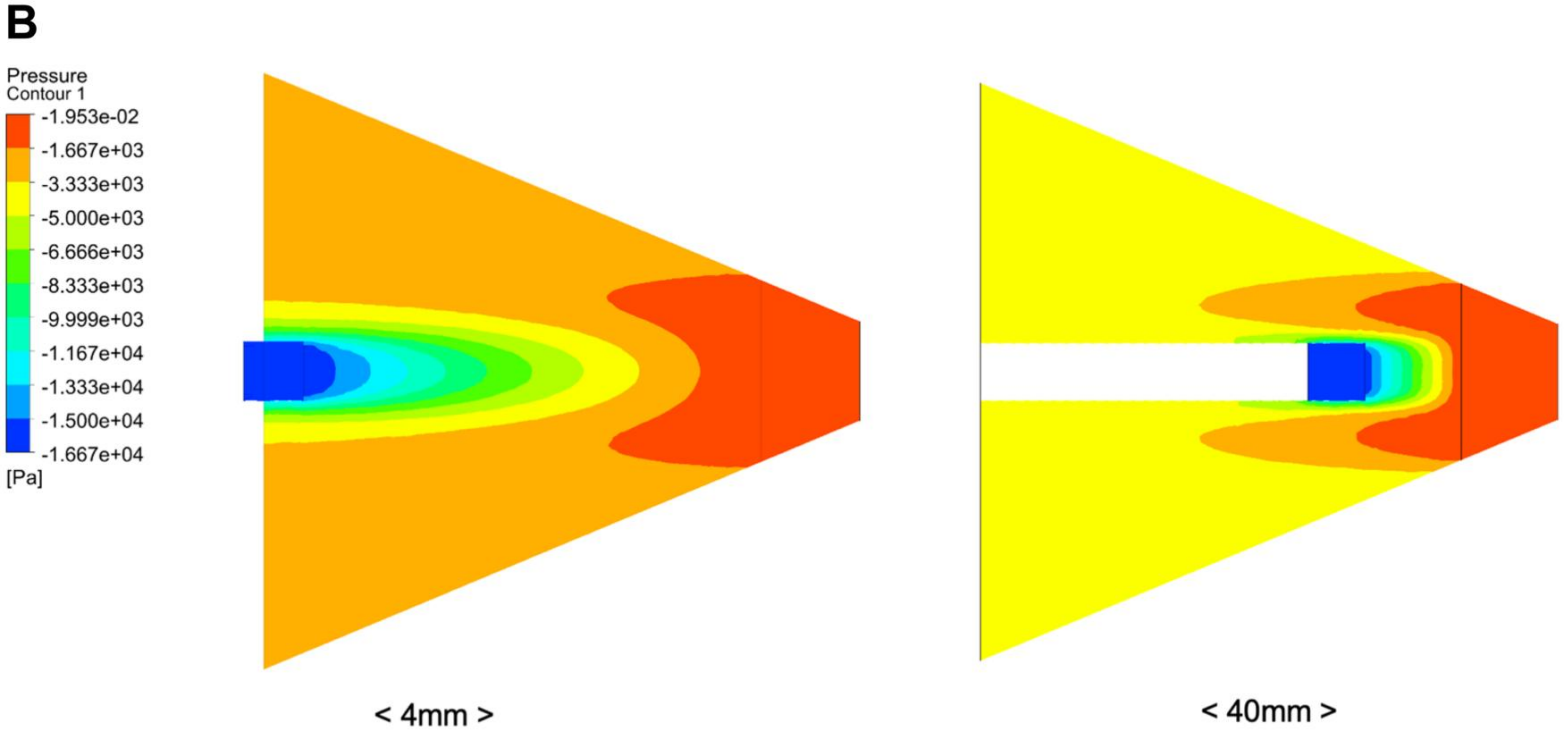
Computational Fluid Dynamics Simulation Showing That the Modified Negative Pressure Wound Therapy Technique Generates Stronger Negative Pressure than the Conventional Method

**Figure 3.**
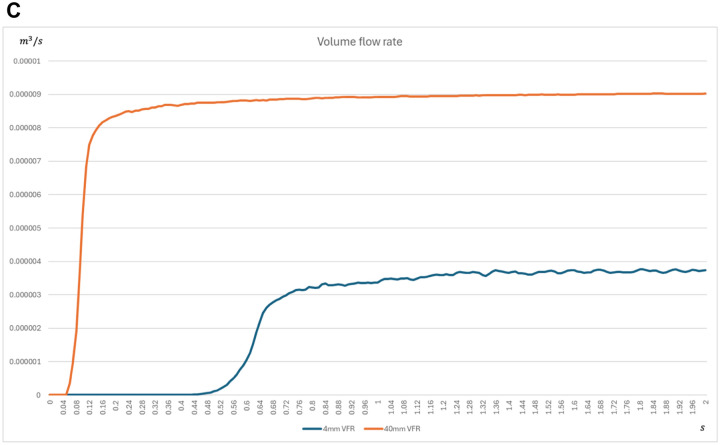
Simulated Volume Flow Rate in the Modified Negative Pressure Wound Therapy Technique Rapidly Peaks and is Maintained at a Level Sufficient to Sustain Consistent Negative Pressure

As illustrated in **Figure 1**, this schematic demonstrates how the modified technique facilitates more efficient delivery of negative pressure to the tissue bed. Computational fluid dynamics simulations further confirmed that this modified NPWT approach produces stronger and more sustained negative pressure compared to the conventional method, with the simulated volume flow rate rapidly peaking and maintaining a sufficient level thereafter (**Figures 2B** and **3**) Experimental data support that this technique improves pressure delivery and enhances the drainage of viscous secretions such as saliva.

Endoscopic vacuum therapy (EVT) achieves favourable outcomes for oesophageal leaks but requires repeated endoscopic exchanges under deep sedation or anaesthesia, adds procedural costs and resource use, and carries anaesthesia-related risk.^6^^,^^7^ For cervical leaks—where the cavity is accessible through the wound—we prefer modified NPWT via the surgical incision; in our experience, it provides continuous drainage and granulation while reducing anaesthetic exposure and direct costs at the bedside. Accordingly, we reserve EVT for intrathoracic defects and manage cervical leaks with modified NPWT.

As with other NPWTs, Haemorrhage is the principal safety concern, particularly if foam or suction contacts exposed vessels.^7^ In our series, leak cavities lay in the deep neck and were separated from the carotid sheath by soft tissue planes, likely buffering suction on major vessels and lowering bleeding risk. When vessels are exposed, the technique should be used with great caution and meticulous sponge positioning; in such cases, alternative strategies may be preferable.

In summary, the modified NPWT technique effectively addresses the limitations of conventional methods in complex anatomical regions. By ensuring a reliable occlusive seal and enhancing negative pressure transmission, this approach promotes optimal wound healing through improved exudate clearance and reduced maceration.

## AUTHORS CONTRIBUTION

Conception and design: Hyo Yeong Ahn; Collection and assembly of data: Ho Seong Cho; Data analysis and interpretation: Min Su Kim and Chiseung Lee; Manuscript writing: Ho Seong Cho and Hyo Yeong Ahn. All authors read and approved the final manuscript.

## Data Availability

The datasets generated and analyzed during the current study are not publicly available due to the privacy of patient data, but are available from the corresponding author on reasonable request and after approval by the Institutional Review Board of the hospital.
